# Comparison of treatment routine using aflibercept: Strict vs. relaxed retreatment regimen (TOLERANT study)—A non‐inferiority, randomized controlled trial

**DOI:** 10.1111/aos.17514

**Published:** 2025-05-06

**Authors:** Damian Jaggi, Lieselotte E. Berger, Sandrine Zweifel, Matthias D. Becker, Stephan Michels, Odile Stalder, Joel‐Benjamin Lincke, Oussama Habra, Sebastian Wolf, Martin S. Zinkernagel

**Affiliations:** ^1^ Department of Ophthalmology, Inselspital, Bern University Hospital University of Bern Bern Switzerland; ^2^ Department of BioMedical Research University of Bern Bern Switzerland; ^3^ Department of Ophthalmology University Hospital Zurich, University of Zurich Zurich Switzerland; ^4^ Spross Research Institute Department of Ophthalmology Zürich City Hospital Zürich Switzerland; ^5^ Department of Ophthalmology University of Heidelberg Heidelberg Germany; ^6^ Augenklinik Zürich West Switzerland; ^7^ CTU Bern University of Bern Bern Switzerland

**Keywords:** aflibercept, AMD, anti‐VEGF, subretinal fluid, tolerance, treat‐and‐extend

## Abstract

**Purpose:**

This trial evaluated the noninferiority of a relaxed compared to a strict treat‐and‐extend treatment strategy in patients with neovascular Age‐related macular degeneration (AMD).

**Methods:**

Multicenter, randomized, controlled, phase IV, non‐inferiority clinical trial. Patients with treatment‐naïve nAMD were randomized 1:1 to a relaxed or strict treat‐and‐extend treatment regimen. Aflibercept 2 mg/0.05 mL was used. In the relaxed regimen, up to 100 μm subfoveal subretinal fluid was tolerated, vs. no tolerance of any fluid in the strict regimen. The primary outcome was the change in best corrected visual acuity (BCVA; ETDRS letters) from baseline to the end of the study at week 104 and its difference between the two treatment arms, with a 5‐letter non‐inferiority margin.

**Results:**

We randomized 150 patients. The full analysis showed non‐inferiority of the relaxed treatment, with a mean difference of −0.12 letters (95%‐CI: −3.45 to infinity, H0; mean. diff. ≤ 5 letters: *p* = 0.008), and a visual acuity gain of 7.3 (4.82; 9.78) vs. 7.01 (3.67; 10.36) letters in the strict vs. relaxed regimen, respectively. Many patients deviated from the protocol due to Covid‐19. Per‐protocol analysis showed a mean difference of −1.78 letters (95%‐CI: −6.61 to infinity, H0; mean. diff. ≤ 5 letters: *p* = 0.136). Fewer injections were needed in the relaxed regimen, with a mean difference of −2.34 (95%‐CI: −4.11 to −0.56, *p* = 0.01).

**Conclusion:**

Tolerating up to 100 μm subfoveal subretinal fluid achieves good visual outcomes in our 24‐month follow‐up period, in patients treated with aflibercept for nAMD, with significantly fewer injections needed.

## 1 INTRODUCTION

The treatment of neovascular age‐related macular degeneration (nAMD) with anti‐vascular endothelial growth factor (anti‐VEGF) agents has revolutionized the field of ophthalmology (Martin et al., 2011; Rosenfeld et al., 2006). Dosing strategies as well as the number of existing agents have increased over the past years. The pivotal trials with monthly dosing have shown high visual acuity gains, but also implied a large treatment burden for patients and healthcare systems (Oubraham et al., 2011; Rosenfeld et al., 2006). In order to address this issue, as‐needed and later treat‐and‐extend (T&E) treatment regimens have been used increasingly (Guymer et al., 2019; Li et al., 2020). While as‐needed (or pro‐re‐nata) regimens include regular clinical visits, treatments are only administered if disease activity is detected at that visit. On the other hand, T&E strategies include an anti‐VEGF injection at every visit but with personalized visit frequencies. T&E strategies have proved the same visual acuity gains as monthly dosing and better gains than as‐needed regimen (Abedi et al., 2014; Rufai et al., 2017). This can be explained by the fact that disease activity is anticipated better by personalized visits. A typical T&E treatment strategy includes 2–3 monthly injections, followed by individualized extension of visit and treatment intervals. In order to provide a successful treatment with a T&E regimen, it is crucial to adequately evaluate disease activity during the extension of patients' visits and treatments. Optical coherence tomography (OCT) findings such as intraretinal fluid (IRF), subretinal fluid (SRF), or change in pigment epithelial detachment (PED), or clinical parameters like macular haemorrhages are therefore usually used. Pivotal trials showed that IRF is a clear sign of activity, as it was associated with poorer outcomes and delayed treatment response (Grechenig et al., 2021; Rofagha et al., 2013). On the other hand, post hoc analyses from the CATT and VIEW studies suggested that SRF might be less severe and tolerated to some extent without losses in visual outcomes (De Massougnes et al., 2018; Jaffe et al., 2018; Schmidt‐Erfurth et al., 2015). These results encouraged the evaluation of treatment approaches where some amount of SRF is tolerated. The FLUID study recently demonstrated noninferiority of such a “relaxed” treatment regimen where SRF was tolerated to up to 200 μm SRF compared to a strict regimen where no fluid was tolerated, in patients with nAMD treated with Ranibizumab (Guymer et al., 2019). Best‐corrected visual acuity (BCVA) gains of the strict vs. relaxed group were 2.6 vs. 3.0 ETDRS letters, respectively. Moreover, participants of the relaxed treatment regimen needed significantly fewer injections (15.8 vs. 17.0) in the FLUID study. The purpose of the here presented trial was to evaluate a similar T&E strategy for aflibercept in patients with newly diagnosed nAMD.

## 2 METHODS

This study was a multi‐center, phase IV, open‐label, two‐arm, parallel‐group, randomized controlled trial, with a duration of two years or 104 weeks. It was conducted in accordance with the international conference on Harmonization of good clinical practice and the Declaration of Helsinki, and local ethics board (KEK Bern No. PB_2016–01603) approval was obtained. Written informed consent was obtained before any study‐related activity. This study is registered at clinicaltrials.gov (NCT02550002).

### 2.1 Randomization, masking/blinding

Patients were randomized 1:1 according to a web‐based system with computer‐generated random numbers prepared by an independent statistician otherwise not involved in the study (CTU Bern standard operating procedure). BCVA assessment staff and the reading centre were blinded to the treatment group. Patients and physicians were not blinded.

### 2.2 Central reading center

A central reading center (Bern Photographic Reading Center, BPRC, Bern, Switzerland) evaluated all retinal imaging. Based on its masked assessment of the OCT images, the retreatment interval was calculated by the central software (RedCap, CTU Bern, University of Bern, Switzerland).

### 2.3 Subjects, inclusion and exclusion criteria

Participants were recruited at four different centres in Switzerland and investigated from December 2015 until February 2022. Participants did not receive any compensation other than reimbursement of travel expenses. Inclusion criteria were diagnosis of nAMD with choroidal neovascularization (CNV) and associated visual impairment. Active nAMD lesions were characterized by SRF and/or IRF and an area of fibrosis less than 50% of the lesion size. CNV membrane was confirmed by the presence of active leakage from the area of CNV seen by fluorescein angiography (FA) and colour fundus photography (CFP) and at least two of the following items: Drusen, RPE‐atrophy, exudation, sub‐ or intraretinal haemorrhage. BCVA, measured on an ETDRS‐Letter chart at baseline, must be 23 letters or more (approximate Snellen‐equivalent: 20/320). Exclusion criteria included: inability to comply with study procedures, pregnant women, severe systemic disease that could bias study assessments, stroke/myocardial infarction within 3 months, hypersensitivity to aflibercept or fluorescein, prior systemic anti‐VEGF use, and other investigational drugs within 6 months. Eye‐specificexclusion criteria included: active inflammation, uncontrolled glaucoma, neovascular glaucoma, intraocular procedure within 2 months, visually significant cataract, aphakia, pseudoexfoliation glaucoma, vitreous haemorrhage, retinal detachment, proliferative vitreoretinopathy, or CNV of any other cause than nAMD, structural damage within 0.5 disc diameter of the fovea, subfoveal subretinal haemorrhage of ≥1 disc diameter, photodynamic therapy, or macular laser therapy.

### 2.4 Intervention/treatment regimen

After randomization, treatment with 2 mg/0.05 mL intravitreal aflibercept (EYLEA®, Regeneron, Tarrytown, NY, USA) was initiated. A visit window of ±3 days was applied for all treatments. Initial injections were applied on baseline and week four. In the relaxed regimen, treatment intervals were then extended by two weeks only if SRF in the central subfoveal field was ≤100 μm in a vertical extent and no IRF was detected on SD‐OCT examination. In the strict regimen, treatment intervals were extended by two weeks only if no SRF and no IRF could be detected. If these “extension criteria” were not met, treatment intervals were shortened by one week until a minimal treatment interval of four weeks was reached. Treatment was administered over a period of 104 weeks.

### 2.5 Examination

BCVA testing was assessed on an ETDRS chart at 4 meters at every visit. Spectral‐domain OCT imaging (Heidelberg Engineering, Heidelberg, Germany) and a standard clinical ophthalmic examination were performed at every visit, as well as a review of consent and safety assessment. Autofluorescence imaging, CFP, and FA were performed at baseline and end of study (EOS).

### 2.6 Outcomes

The primary outcome was defined as the change in BCVA from baseline to EOS at week 104 and its difference between the two treatment arms. Secondary outcomes were this same outcome at week 52, as well as the difference between the two arms in the mean change of the following (all from baseline to week 52 and EOS): central subfield thickness (CST), number of injections, proportion of patients showing no IRF and SRF, and proportion of patients showing no SRF. Other secondary outcomes were: the time course of BCVA, the number and percentage of patients per group with improvement of BCVA (≥5, ≥10, and ≥15 letters), with loss of BCVA (<−5, <−10, and < −15 letters), with BCVA ≥73 letters (20/40 Snellen equivalent), with dry retina, with CST ≤300, ≤250, and ≤200 μm, and effectiveness of the two regimens over time on BCVA and CST based on number of injections required from baseline to EOS.

### 2.7 Safety outcomes

We assessed the total incidence as well as the difference between the two arms in the number of ocular and general adverse events (AE) and serious adverse events (SAE).

### 2.8 Determination of sample size/power analysis

The sample size calculation was based on the following assumptions: for a non‐inferiority design and assuming a standard deviation of the mean change from baseline to month 24 of 12, with a non‐inferiority margin of 5 letters, a total of 72 patients per group will be required to achieve 80% power at a one‐sided 5% significance level. We will include 75 patients per group to allow for some drop‐outs.

### 2.9 Statistical analysis

Categorical variables were presented as frequencies and percentages; continuous variables were presented as medians with interquartile range [IQR]. All analyses were done in Stata version 17.0 (StataCorp. Stata Statistical Software: Release 17. StataCorp, College Station, TX: StataCorp LP., 2021.). Continuous outcomes were analysed using mixed‐effects linear models with the treatment group and the baseline value as fixed effects and a random intercept for centre. For the primary outcome, the mean difference between groups with a one‐sided 95%‐CI and a *p*‐value for non‐inferiority (using the margins of 5 units) was presented, and conventional two‐sided 95%‐CI and its *p*‐values for secondary outcomes were displayed. Missing values were imputed following the last value carried forward principle. We also ran multiple imputations and the data sets were analysed and described using Rubin's rules to combine results across data sets. Binary secondary outcomes were analysed using Mantel–Haenszel risk difference. Repeatedly measured outcomes were analysed using mixed‐effects linear or logistic models. The same models were used to estimate the association with the number of injections. The area under the curve was computed under the linear fitted line from the previous mixed‐effects models, from baseline until 24months but adjusted for baseline values.

The primary analysis was done on the full analysis set (FAS) including all randomized patients in adherence to the intention‐to‐treat principle; a secondary analysis was done on the per‐protocol (PP) set. This analysis excluded patients with a delay of more than five days from the planned visits according to the protocol. Safety analysis summarizing the adverse events was shown in tables as descriptive analysis.

## 3 RESULTS

**FIGURE 1:**
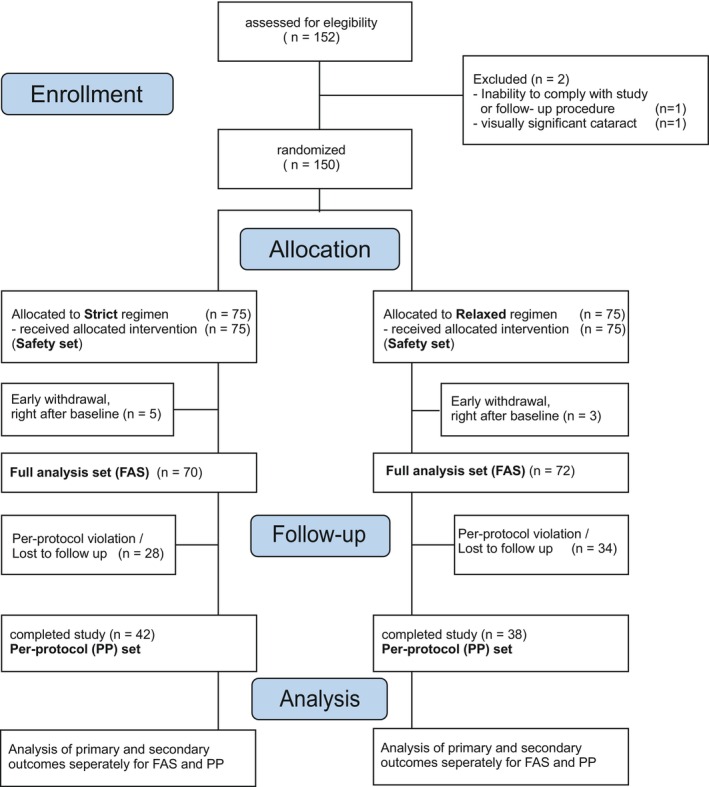
Consolidated Standards of Reporting Trials (CONSORT) flow diagram.

A total of 150 patients were eligible and randomized (75 to each treatment arm). Eight patients (five in the strict, 3 in the relaxed regimen) had to be excluded from the primary analysis, all due to early consent withdrawal right after baseline. The remaining patients (strict group, *n* = 70; relaxed group, *n* = 73) were the FAS. Of these, 62 patients had a deviation from the study protocol to one or a combination of the following: visits out of time‐window (20), BCVA EOS incomplete (35), early discontinuation of study (11), EOS invalid time window (2), No IRF/SRF at baseline (2), discontinuation or out‐of‐window of aflibercept injections before week 96 (50). Reasons included consent withdrawal (28), death (8), treatment continued at private practitioner (6) poor general condition (4), and other (4). Hence, 80 patients remained in the PP analysis (strict group, *n* = 42; relaxed group, *n* = 38). A standardized study flow diagram is presented in Figure 1. Sensitivity analysis from multiple imputation as well as join‐model analysis is provided as Supporting Infomation.

### 3.1 Baseline characteristics

Generally, baseline characteristics were well balanced between the two groups, except for Geographic atrophy (GA), which was more frequent in the relaxed group. Median age was 79 [74, 85] years, 88 (59%) of the patients were female, a BMI of 25 [23, 28]. Median BCVA was 65 [57, 75] for the study eye, and 78 [60, 85] for the fellow eye. Intraocular pressure was 15 [13, 16], lens status was phakic for 73 (49%) and pseudophakic for 77 (51%) of the patients. OCT grading showed that CST was 398 [336, 459] μm, IRF was present in 112 (75%), SRF was present in 106 (71%), where median SRF was 94 [52, 155] μm. PED was seen in 48 (32%) of the patients. Detailed baseline characteristics are presented in Table 1.

**TABLE 1:** Demographics and baseline characteristics.

Category		Total (*N* = 150)	Strict regimen (*N* = 75)	Relaxed regimen (*N* = 75)	*p*‐value
Age at baseline	[years]—median [lq, uq]	79 [74, 85]	81 [75, 84]	79 [74, 85]	0.74
Gender	Female	88 (59%)	43 (57%)	45 (60%)	0.87
	Male	62 (41%)	32 (43%)	30 (40%)	
BCVA	Study eye—median [lq, uq]	65 [57, 75]	65 [56, 72]	65 [58, 75]	0.62
	Fellow eye—median [lq, uq]	78 [60, 85]	77 [60, 84]	78 [56, 85]	0.58
IOP	[mmHg]—median [lq, uq]	15 [13, 16]	15 [12, 16]	15 [13, 16]	0.71
Lens	Phakic	73 (49%)	34 (45%)	39 (52%)	0.51
	Pseudophakic	77 (51%)	41 (55%)	36 (48%)	
	Aphakic	0 (0%)	0 (0%)	0 (0%)	
	No opacity	2 (2.7%)	0 (0%)	2 (5.1%)	0.61
	Mild to moderate opacity	34 (47%)	16 (47%)	18 (46%)	
	Severe opacity	37 (51%)	18 (53%)	19 (49%)	
Drusen	No	1 (0.67%)	0 (0%)	1 (1.3%)	1.00
	Yes	149 (99%)	75 (100%)	74 (99%)	
Exudates	No	99 (66%)	48 (64%)	51 (68%)	0.73
	Yes	51 (34%)	27 (36%)	24 (32%)	
Haemorrhages	No	62 (41%)	33 (44%)	29 (39%)	0.62
	Yes	88 (59%)	42 (56%)	46 (61%)	
PED	No	102 (68%)	49 (65%)	53 (71%)	0.60
	Yes	48 (32%)	26 (35%)	22 (29%)	
GA	No	130 (87%)	67 (89%)	63 (84%)	0.33
	Yes	20 (13%)	8 (11%)	12 (16%)	
	Area [mm^2^]—median [lq, uq]	0.59 [0.09, 1.8]	1.5 [0.59, 1.8]	0.19 [0.08, 2.3]	0.39
CST	[μm]—median [lq, uq]	398 [336, 459]	403 [349, 457]	394 [321, 490]	0.56
IRF	No	38 (25%)	20 (27%)	18 (24%)	0.85
	Yes	112 (75%)	55 (73%)	57 (76%)	
SRF	No	44 (29%)	22 (29%)	22 (29%)	1.0
	Yes	106 (71%)	53 (71%)	53 (71%)	
	[μm]—median [lq, uq]	94 [52, 155]	103 [52, 155]	89 [59, 155]	0.56

Abbreviations: BCVA, best corrected visual acuity in letters; CST, central subfield thickness; GA, geographic atrophy; IOP, intraocular pressure; IRF, intraretinal fluid; PED, pigment epithelial detachment; SRF, subretinal fluid.

### 3.2 Primary outcome

The FAS analysis showed a mean improvement of BCVA of 7.30 (95%‐CI: 4.82 to 9.78, *n* = 70) letters in the strict, and 7.01 (95%‐CI: 3.67 to 10.36, *n* = 72) letters in the relaxed regimen, with a mean difference between the two groups of −0.12 (one‐sided 95%‐CI: −3.45 to infinity) letters, and a *p*‐value for H0; mean difference ≤ −5 letters, *p* = 0.008. This leads to the rejection of the null hypothesis, demonstrating noninferiority of the relaxed regimen in the FAS set, as presented in Table 2. Superiority analysis showed that neither set was superior to another, with a mean difference of −0.12 (two‐sided 95%‐CI: −4.09 to 3.86) letters. Analysis of the PP set showed a mean improvement of BCVA of 7.10 (95%‐CI: 3.48 to 10.71, *n* = 42) letters in the strict, and 5.03 (95%‐CI: 0.00 to 10.05, *n* = 38) letters in the relaxed regimen, with a mean difference between the two groups of −1.78 (one‐sided 95%‐CI: −6.61 to infinity) letters, and a *p*‐value for H0; mean difference ≤ −5 letters, *p* = 0.136. Hence, noninferiority cannot be demonstrated in the PP set.

**TABLE 2:** Primary outcome.

Non‐inferiority, full analysis set				
	Strict (*n* = 70)	Relaxed (*n* = 72)	Relaxed—strict	*p*‐value
	Mean (95% ‐ CI)	Mean (95% ‐ CI)	Mean diff. (one‐sided 95% ‐ CI)	
Study eye BCVA (end of study—baseline)	7.30 (4.82, 9.78)	7.01 (3.67, 10.36)	−0.12 (−3.45 to infinity)	0.008*

Superiority, full analysis set				
	Strict (*n* = 70)	Relaxed (*n* = 72)	Relaxed—strict	
	Mean (95% ‐ CI)	Mean (95% ‐ CI)	Mean diff. (two‐sided 95% ‐ CI)	
Study eye BCVA (end of study—baseline)	7.30 (4.82, 9.78)	7.01 (3.67, 10.36)	−0.12 (−4.09 to 3.86)	0.954

Non‐inferiority, per‐protocol set				
	Strict (*n* = 42)	Relaxed (*n* = 38)	Relaxed—strict	
	Mean (95% ‐ CI)	Mean (95% ‐ CI)	Mean diff. (one‐sided 95% ‐ CI)	
Study eye BCVA (end of study—baseline)	7.10 (3.48, 10.71)	5.03 (0.00, 10.05)	−1.78 (−6.61 to infinity)	0.136*

Superiority, per‐protocol set				
	Strict (*n* = 42)	Relaxed (*n* = 38)	Relaxed—strict	
	Mean (95% ‐ CI)	Mean (95% ‐ CI)	Mean diff. (one‐sided 95% ‐ CI)	
Study eye BCVA (end of study—baseline)	7.10 (3.48, 10.71)	5.03 (0.00, 10.05)	−1.78 (−7.53 to 3.98)	0.545

*Note*: Missing observations were imputed following the last value carried forward procedure. Adjusted for baseline BCVA value.

Abbreviations: BCVA, best corrected visual acuity; CI, confidence interval.

* *p*‐value for non‐inferiority with H0: mean difference ≤ −5.

### 3.3 Secondary outcomes on visual acuity

**FIGURE 2:**
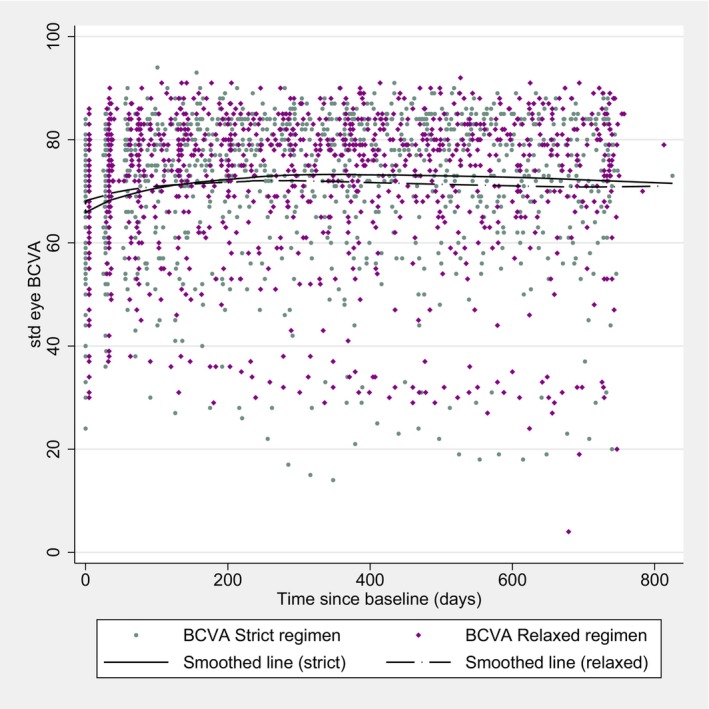
Best‐corrected visual acuity in standard ETDRS letters.

No significant difference in BCVA gains was found at week 52 in the FAS set, with 7.95 vs. 7.74 letters (*p* = 0.98), in the strict vs. relaxed regimen, respectively. An outline of the BCVA over the complete study period is shown in Figure 2. The analysis of the PP set neither showed significant differences at week 52, with 8.20 vs. 6.92 letters (*p* = 0.70). The Proportions of participants with different gains or losses from baseline to EOS did not differ between the two groups in the FAS. Fifty‐four (77%) and 52 (72%) patients showed a gain in BCVA (>0 letters), in the strict and relaxed regimen, respectively (*p* = 0.51). Comparable results were found in the groups of BCVA gains of ≥5 letters (59%, vs. 54%, *p* = 0.57), ≥10 letters (43% vs. 47%, *p* = 0.62) and ≥15 letters (21% vs. 28%, *p* = 0.38). Proportions of BCVA reduction showed the following: < −5 letters (9% vs. 14%, *p* = 0.33), < −10 letters (4% vs. 10%, *p* = 0.21), and < −15 letters (4% vs. 10%, *p* = 0.73), in the strict vs. relaxed regimen, respectively. Mixed‐effect models demonstrate noninferiority at 12, but not at 24 months (mean difference of −0.29 letters, one‐sided 95%‐CI: −2.58 to infinity, at 12 months; mean difference of −1.69 letters, one‐sided 95%‐CI: −5.46 to infinity).

### 3.4 Number of injections

**FIGURE 3:**
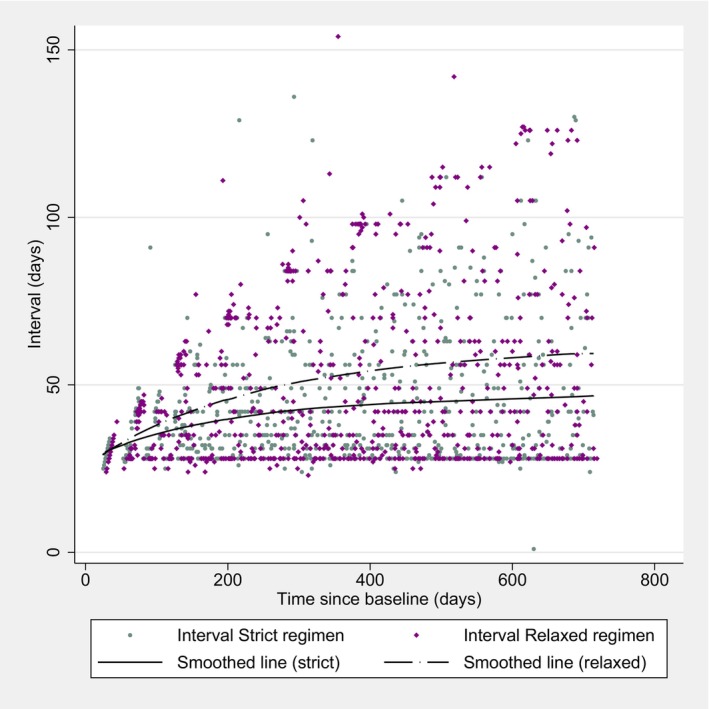
Injection intervals over time.

A mean total of 17.6 vs. 15.3 injections were administered in the strict vs. relaxed regimen, respectively, with a mean difference of −2.34 (95%‐CI: −4.11 to −0.56, *p* = 0.010). A significant difference between the two groups was also found at week 52, where 10.2 vs. 9.2 injections were administered, with a mean difference of −0.98 (95%‐CI: −1.90 to −0.05, *p* = 0.038). The fastest extension of the intervals happened in the first year of treatment, as shown in Figure 3.

### 3.5 Intra‐ and subretinal fluid and central retinal thickness

Both groups showed a good overall reduction in CST over the two years period. There was a reduction of 121.9 vs. 145.3 μm in the strict vs. relaxed regimen, with a mean difference of −23.4 μm (95%‐CI: −44.6 to −2.1 μm, *p* = 0.03) at the EOS. No significant difference was found at week 52, with a reduction of 123.0 vs. 125.9 μm, with a mean difference of −2.4 μm (95%‐CI: −23.8 to 19.0 μm, *p* = 0.83). The proportion of participants with IRF decreased from 73% to 26% under the strict regimen and from 76% to 21% under the relaxed regimen. The proportion of participants with SRF decreased from 71% to 23% under the strict regimen and from 71% to 14% under the relaxed regimen. No significant difference in IRF/SRF was found between the two groups at any time. However, there was a significant difference in GA, with a larger proportion in the relaxed regimen (27% vs. 47%, risk difference: 0.20, *p* = 0.016) at EOS and week 52 (19% vs. 36%, risk difference: 0.18, *p* = 0.022).

### 3.6 PED—Subgroup analysis

Patients with PED were analysed in a subgroup for the primary and secondary outcomes. The FAS analysis showed a mean improvement of BCVA of 5.80 (95%‐CI: 0.87 to 10.73, *n* = 25) letters in the strict, and 4.43 (95%‐CI: −3.54 to 12.40, *n* = 21) letters in the relaxed regimen, with a mean difference between the two groups of 1.17 (one‐sided 95%‐CI: −5.35 to infinity) letters, and a p‐value for H0; mean difference ≤ −5 letters, *p* = 0.060. No significant difference was found at week 52 either (mean difference = 0.07, *p* = 0.98). Comparable results were found in the groups of BCVA gains of ≥5 letters (40%, vs. 48%, *p* = 0.55), ≥10 letters (32% vs. 38%, *p* = 0.69) and ≥15 letters (24% vs. 33%, *p* = 0.38), in the strict vs. relaxed regimen, respectively. CST reduction was less in the strict group with −104.2 vs. −171.2 μm, with a mean difference of −65.2 μm (95%‐CI: −114.6 to −15.9 μm, *p* = 0.01). The proportion of SRF presence at the EOS was 28% vs. 19% in the strict vs. relaxed group (*p* = 0.48). The total number of injections was significantly higher in the strict group, with 19.08 vs. 15.71 (95%‐CI: −6.38 to −0.35, *p* = 0.029) injections. Due to the small sample size and high variability in the PP set, statistics were limited to the FAS.

### 3.7 Safety outcomes

No difference between the groups regarding AE's and SAE's could be detected. No new adverse events for aflibercept use were reported.

## 4 DISCUSSION

In this randomized controlled trial, we presented data on visual and anatomical outcomes in patients treated with aflibercept for newly diagnosed nAMD. Usually, treatment of nAMD with any available anti‐VEGF agent aims at drying the retina, or at least central macula, completely. Our two‐treatment regimen differed only in the strategy of tolerating up to 100 μm subfoveal SRF and showed both good visual acuity progress over the observed time. FAS analysis demonstrated noninferiority of the relaxed compared to the strict treatment arm, but per‐protocol analysis did not. The PP‐analysis is based on a sample size nearly half as small as the FAS set. This loss of sample size in the PP‐set was mainly owed to deviations from the study protocol due to the COVID‐19 pandemic. These deviations of the study protocol, as well as the imputation by the “last observation carried forward” strategy in the FAS can cause a certain effect dilution of the data (estimated difference is shifted towards zero). This can also lead to falsely rejection of the null hypothesis. However, the two groups differed significantly in the number of injections, whereby the relaxed treatment arm received only 14.8 injections compared to 18.6 injections in the strict arm. This finding is remarkable and is in keeping with the recently published FLUID study by Guymer et al., which tolerated up to 200 μm subfoveal SRF, in patients treated with Ranibizumab (Guymer et al., 2019). There, the strict vs. relaxed arm received 17.0 vs. 15.8 injections, respectively. The visual acuity improvement of the here presented TOLERANT study with 7.30 letters in the strict, vs. 7.01 letters in the relaxed arm is also noteworthy and is in accordance with the pivotal nAMD studies (CATT, VIEW, and FLUID [4.0 vs. 4.3 letters]) (Jaffe et al., 2018). Furthermore, we explored the nature of these patients who discontinue the treatment prematurely through a joint model (post‐hoc analysis) and its results do not show evidence informative drop‐outs, which support the longitudinal analysis through the mixed‐effects linear regression of the longitudinal data without imputation. This model supports the PP‐set analysis, which cannot reject the inferiority of the relaxed regimen. The PP‐set showed a trend in favour of the strict‐regimen in terms of visual acuity outcome (not significant). Our anatomical data shows a general good reduction of the CST and reduction of SRF/IRF over time. However, it is not quite clear, how the significantly greater reduction in CST in the relaxed group can be explained. One explanation could be that there is a connection with the also significantly higher proportion of GA in the relaxed group. Since areas of GA show a thinner retinal thickness due to absence of outer retinal layers, the higher proportion of GA in the relaxed group would cause this finding. Another explanation could be that there is only statistical significance because of multiple testing, and the measured difference would be within the null hypothesis. Moreover, the higher proportion of GA could best be explained due to the random imbalance of GA distribution already at baseline, since injection frequency and regimen has shown to not affect macular atrophy incidence or progression (Spooner et al., 2020). The PED patients needed significantly fewer injections in the relaxed compared to the strict group, with comparable visual outcomes. This adds to the hypothesis that SRF in the presence of PED might occur due to RPE‐malfunction rather than active MNV (Hosseini et al., 2021) and to findings that suggested SRF‐tolerance in non‐neovascular PED patients (Cho et al., 2023). Since the PED subgroup included only 32% of the patients, the variability was much higher and the results need to be interpreted cautiously.

In general, our results show that both treatment regimens show functional and anatomic results that are comparable with the aforementioned trials, with a good safety profile. However, recent post‐hoc analysis from HAWK and HARRIER trials showed that the absence of any retinal fluid at more visits had a positive effect on visual and morphologic outcomes (Eichenbaum et al., 2023). A recent meta‐analysis suggested that the presence of SRF still might be a protective factor and was associated with slightly better BCVA at the last study observation (Patil et al., 2022). Clearly, further investigation and individualized protocols will be needed to gain further insight in this field.

### 4.1 Limitation

The study has several limitations. The main limitation is the large number of dropouts due to protocol deviations due to the COVID pandemic. The power calculation used a one‐sided alpha of 5%, which was common at the time of the study design in 2015. Nowadays there is a consensus that an alpha of 2.5% would be more accurate in non‐inferiority trials. Furthermore, we had calculated too few dropouts. Another limitation is the heterogeneity of the study population in terms of GA distribution, probably related to the small sample size, and that CNV was not classified according to more recent Grading (MNV Type 1–3) (Spaide et al., 2020).

## 5 CONCLUSION

In aflibercept‐treated nAMD patients, tolerating up to 100 μm SRF yields good visual outcomes with fewer injections. However, the results have to be interpreted carefully since many patients were lost to follow up during Covid‐19.

## FUNDING INFORMATION

The study was financially supported by Bayer (Schweiz) AG. Bayer had no role in the design or conduct of this research.

## FINANCIAL DISCLOSURE

Damian Jaggi, Liselotte Berger, Odile Stalder, Joel Lincke, Oussama Habra: None. Novartis, Alcon, Appelis, Bayer, Endogena, Zeiss, Roche: Sandrine Zweifel. W.H. Spross Stiftung der Förderung der Augenheilkunde, Roche, Novartis, Chengdu Kanghong, Stiftung Stadtspital Zürich, Hoffmann—La Roche Ltd., Zeiss meditec (Germany), Oertli (Switzerland), Orthorobotics AG: Matthias Becker. Bayer, Roche, Novartis, Apellis, Ophthorobotics: Stephan Michels. Allergan, Bayer, Novartis, Heidelberg Engineering, Hoya, Optos, Euretina: Sebastian Wolf. Allergan, Bayer, Novartis, Heidelberg Engineering, Boehringer Ingelheim: Martin S. Zinkernagel.

## Supporting information


**Appendix S1:** Supporting Information.

**Appendix S2:** Supporting Information.
