# Supplementary material for: Comparison of treatment routine using aflibercept: Strict vs. relaxed retreatment regimen (TOLERANT study)—A non‐inferiority, randomized controlled trial
Source: Acta Ophthalmol. 2025 May 6;103(6):e385–93. doi: 10.1111/aos.17514 (PMC12340175; doi:10.1111/aos.17514)
Supplement: Supplementary file 1 — Appendix S1: Supporting Information. [file AOS-103-e385-s002.docx]

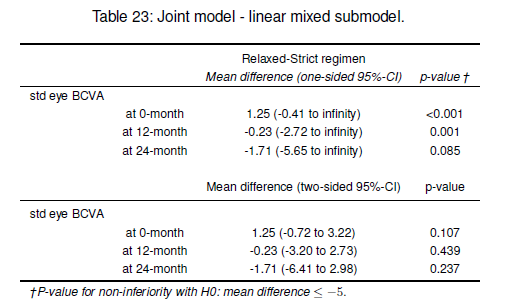


Joint model (JM) analysis was done to assess the presence of informative drop-out. JM combines both the linear mixed model and the Cox model, allowing for estimation of the effect of BCVA on drop-out. BCVA measured over time was the outcome in the linear mixed model, furthermore BCVA measured over time enters also the JM as a predictor for drop-out alongside the other predictors from the Cox submodel. Linear mixed submodel for longitudinal BCVA measurements:

Linear mixed-effects model based on the model in Part 5.3.2 (p.14), excepted the inclusion of center as random intercept.


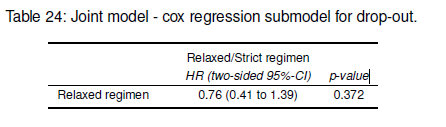


In comparison to the strict regimen, relaxed regimen patients have a non-significant 0.76-fold risk of

drop-out in this model. Moreover, the JM shows no evidence of association between linear mixed submodel and Cox submodel (association coef. -.009, p-value 0.277). Hence, there is no evidence for informative drop-out, i.e. the drop-out does not influence the longitudinal data (BCVA measurements over time) analysis.
