# Supplementary figures and images for: Comparison of treatment routine using aflibercept: Strict vs. relaxed retreatment regimen (TOLERANT study)—A non‐inferiority, randomized controlled trial
Source: Acta Ophthalmol. 2025 May 6;103(6):e385–93. doi: 10.1111/aos.17514 (PMC12340175; doi:10.1111/aos.17514)

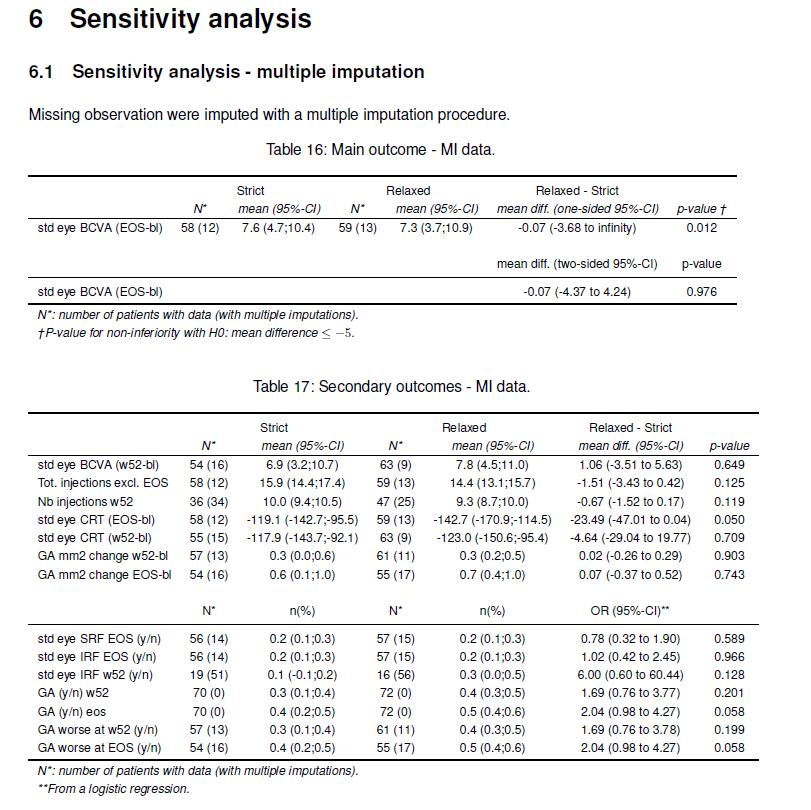

Supplement: Supplementary file 2 — Appendix S2: Supporting Information. [file AOS-103-e385-s001.docx]
